# Supplementary material for: aPKC controls endothelial growth by modulating c-Myc via FoxO1 DNA-binding ability
Source: Nat Commun. 2018 Dec 17;9:5357. doi: 10.1038/s41467-018-07739-0 (PMC6297234; doi:10.1038/s41467-018-07739-0)
Supplement: Supplementary file 1 — Supplementary Information [file 41467_2018_7739_MOESM1_ESM.pdf]

Supplementary Information:

Riddell et al.

aPKC controls endothelial growth by modulating c-Myc via FoxO1 DNA binding ability

Supplementary Figure 1

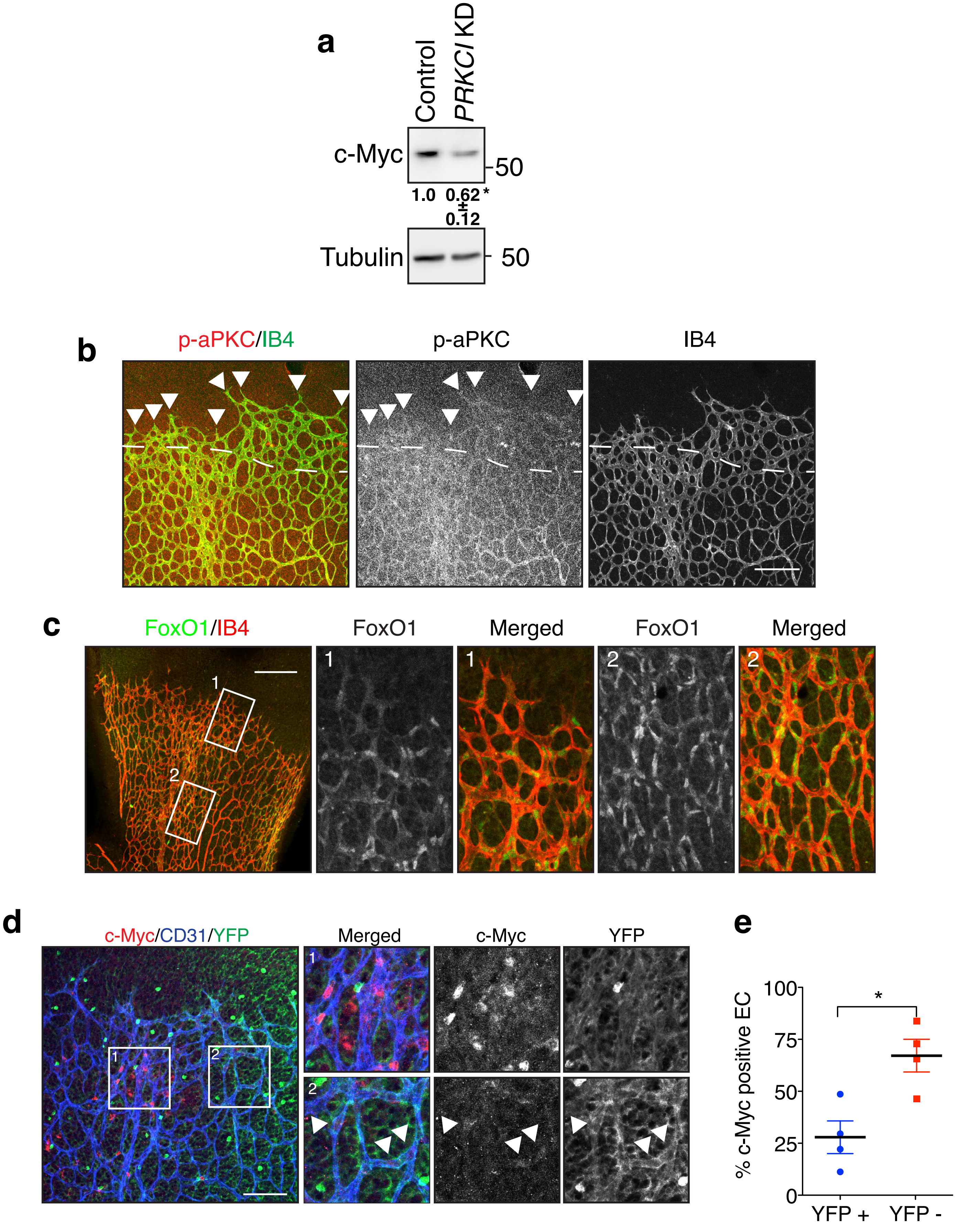

Supplementary Figure 1: **(a)** Western blot analysis of c-Myc in HUVEC treated with siRNA targeting *PRKCI*; Densitometric quantifications are shown below the lanes mean +/- S.E.M.; two-tailed students t-test; *=p<0.05; (*n*=4). (**b**) Representative image of activated phospho-aPKC (p-aPKC) in the P6 retina. Dashed line indicates approximate threshold between high and low p-aPKC signal. Arrowheads indicate the location of tip cells. Images are representative of three animals examined. Scale bar represents 150 μm. **(c)** Representative composite tile-scanned image of FoxO1 and IB4 in control P6 retina. Right panels show higher magnification images of indicated regions. (1) angiogenic front (2) vascular plexus. Images are representative of >three animals examined. Scale bar represents 200 μm. **(d)** Representative image of P6 retinal vascular front after a low dose tamoxifen injection at P1 to induce mosaic *Prkci* $^{i\Delta EC}$ deletion using a Cre inducible EYFP reporter mouse. c-Myc (red) CD31 (blue) EYFP (green). Right panels show higher magnification images of indicated EYFP negative (1) and EYPF positive (2) regions. Arrowheads indicate EYFP positive c-Myc positive cells. Images are representative of 4 animals examined. Scale bar represents 100 μm. **(e)** Percentage of EC expressing c-Myc per field in mosaic deletion P6 retina vascular front; control (YFP-) versus aPKC KO (YFP+); two-tailed students t-test; *=p<0.05; (*n*=4).

Riddell et al., Supplementary Fig. 2

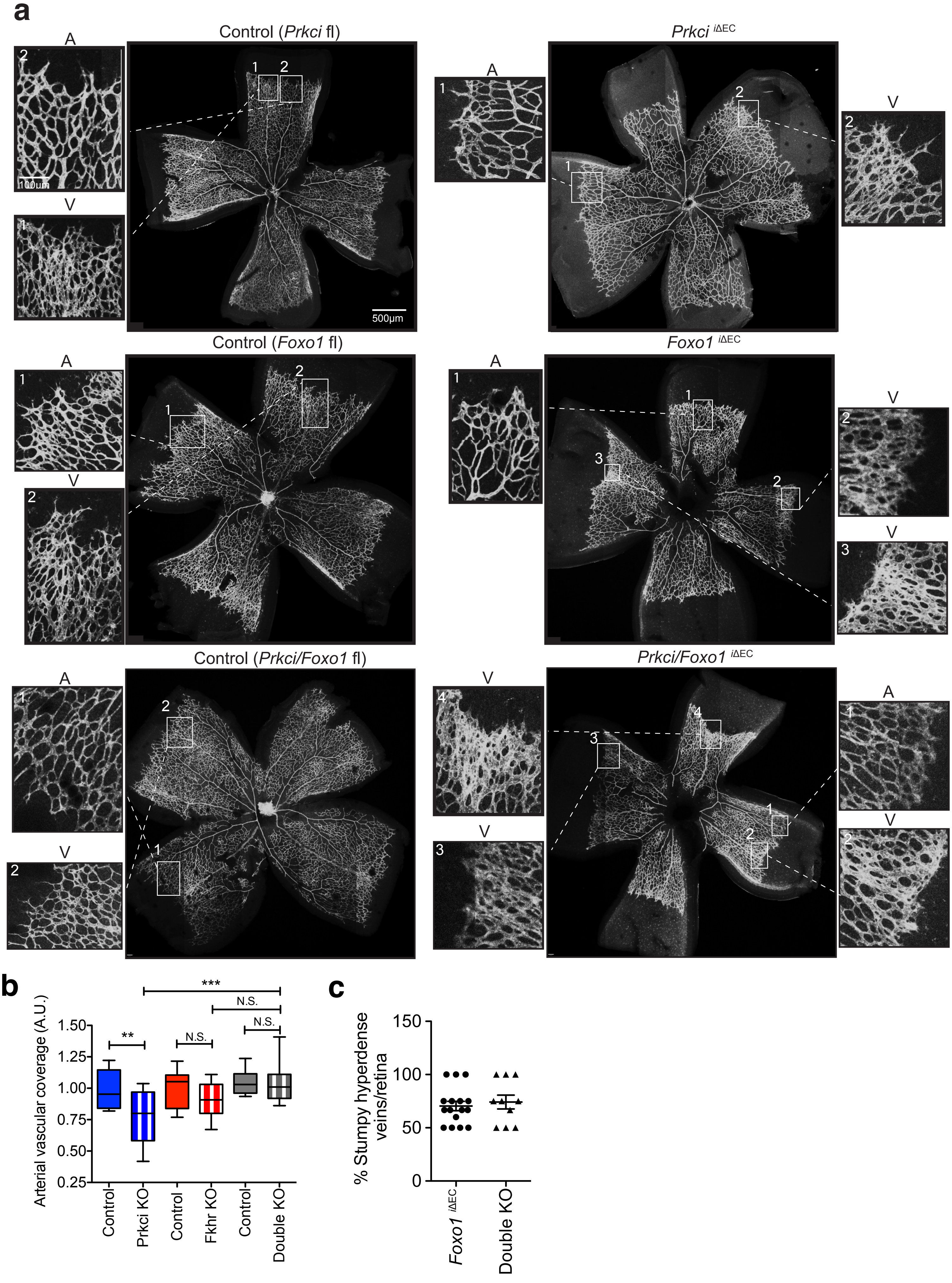

Supplementary Figure 2: **(a)** Composite confocal images of tilescanned IB4 stained P6 retinal vasculature of littermate control and *Prkci* $^{i\Delta EC}$, *FoxO1* $^{i\Delta EC}$, and *Prkci*/*FoxO1*$^{i\Delta EC}$. Boxes indicate highlighted areas. A=arterial region; V= venous region. Images are representative of ≥10 animals of each genotype. Scale bar represents 500 µm. **(b)** Quantification of relative ratio of vascular coverage in the arterial region; line represents median, whiskers representing range; one-way ANOVA Bonferroni post hoc analysis; n.s=p>0.05; **=p<0.01; ***=p<0.001; (*n*≥10). **(c)** Quantification of stumpy hyperdense venous regions observed in *FoxO1* $^{i\Delta EC}$ and *Prkci*/*FoxO1*$^{i\Delta EC}$ retinas. Line represents median value +/- S.E.M. (*n*≥10).

Riddell et al., Supplementary Fig. 3

a

Positions within proteins	Intensity				sequence
	GST	GST-FoxO1	GST-ATP+aPKC	GST-FoxO1ATP-+aPKC	
164	0	0	3,93E+05	5,87E+07	NAWGNLS(1)YADLITK
212	0	0	0	2,81E+07	DKGDSNSSAGWKNS(1)IR
218	0	0	9,24E+05	2,47E+08	HNLS(1)LHSK
221	0	0	0	3,73E+07	HNLSLHS(1)K
323	0	0	0	6,00E+06	TSSNAS(0.169)T(0.815)IS(0.016)GR
329	0	0	0	0	TSSNASTISGRLS(1)PIMTEQDDLGEGDVHSMVYPPSAAK
235	0	0	0	0	VQNEGTGKS(0.249)S(0.751)WWMLNPEGGK
261	0	0	0	9,27E+06	AASMDNNS(1)KFAK

b

FoxO1	(209)	WKNSIRHNLSLHSKFIRVQ	(227aa)
FoxO3	(206)	WKNSIRHNLSLHSRFMRVQ	(224aa)
FoxO4	(146)	WKNSIRHNLSLHSKFIKVH	(165aa)
FoxO6	(137)	WKNSIRHNLSLHTRFIRVQ	(155aa)
dFoxO	(144)	WKNSIRHNLSLHNRFMRVQ	(162aa)
DAF-16	(224)	WKNSIRHNLSLHSRFMRIQ	(242aa)

c

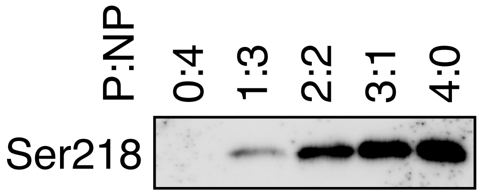

d

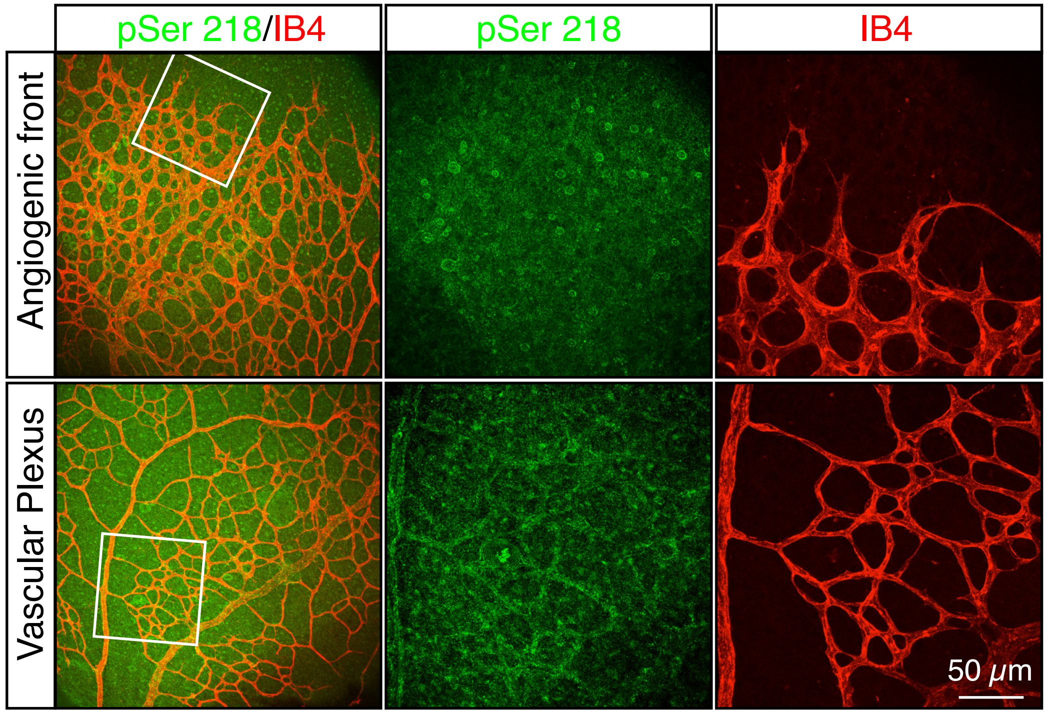

Supplementary Figure 3: (a) aPKCλ phosphorylation sites of FoxO1 identified by mass-spectrometry analysis. Specific scores and detected sequence windows are shown. GST-FoxO1 was phosphorylated by purified aPKCλ in the presence of ATP. GST alone or GST incubated with the kinase and ATP were used as negative controls. **(b)** Amino acid sequence of mammalian FoxO proteins and FoxO homologs in *D. melanogaster* (dFoxO) and *C.* elegans (DAF-16) surrounding the identified phosphorylation site. **(c)** GST-FoxO1 DBD containing the indicated ratio of FoxO1 DBD *in vitro* phosphorylated by aPKCλ to non-phosphorylated FoxO1 DBD as detected by an anti-pSer218 FoxO1 antibody. P:NP; ratio of phosphorylated-protein to non-phosphorylated protein. **(d)** Phosphorylation of FoxO1 at Ser218 in the P6 retinal vasculature as detected with the second anti-pSer218 FoxO1 antibody (green). Isolectin B4 is shown as red signal. Images are representative of signal observed in 3 animals. Scale bar represents 50 μm.

Riddell et al., Supplementary Fig. 4

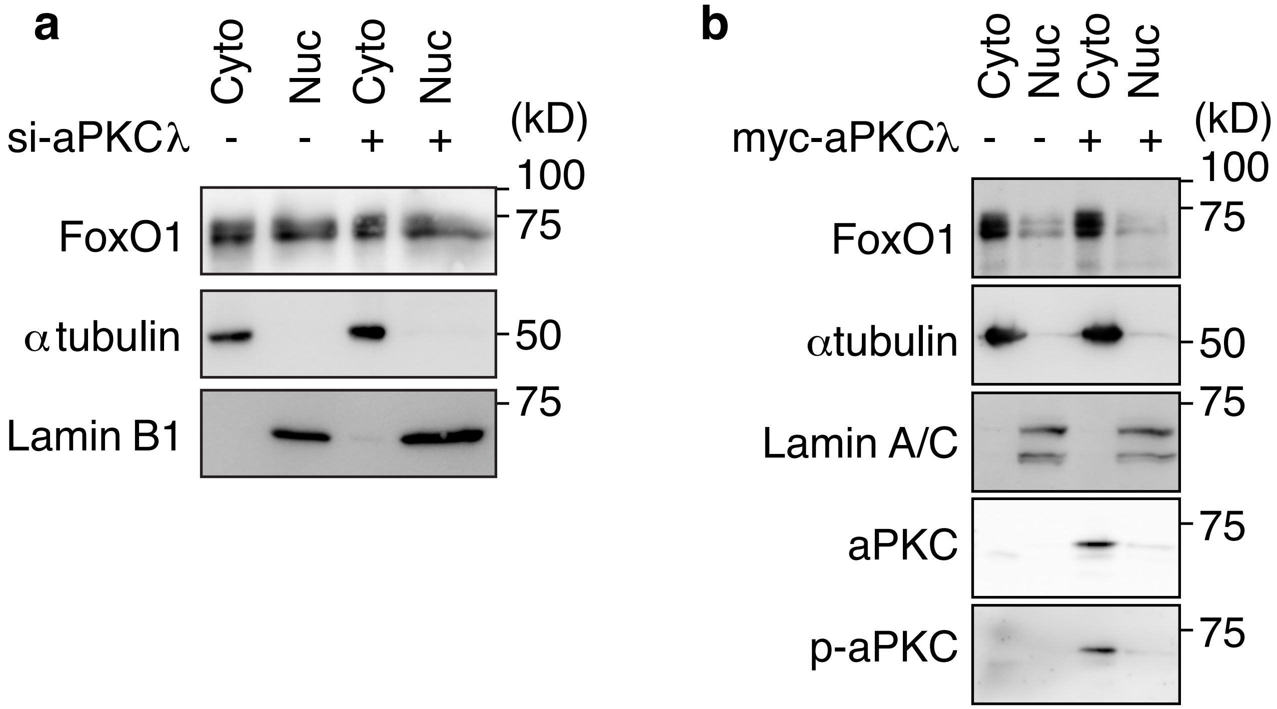

Supplementary Figure 4: Effect of aPKCλ KD (**a**) or aPKCλ over-expression **(b)** on FoxO1 cellular localization as examined with nuclear fractionations and subsequent western blot analysis.

Supplementary Figure 5

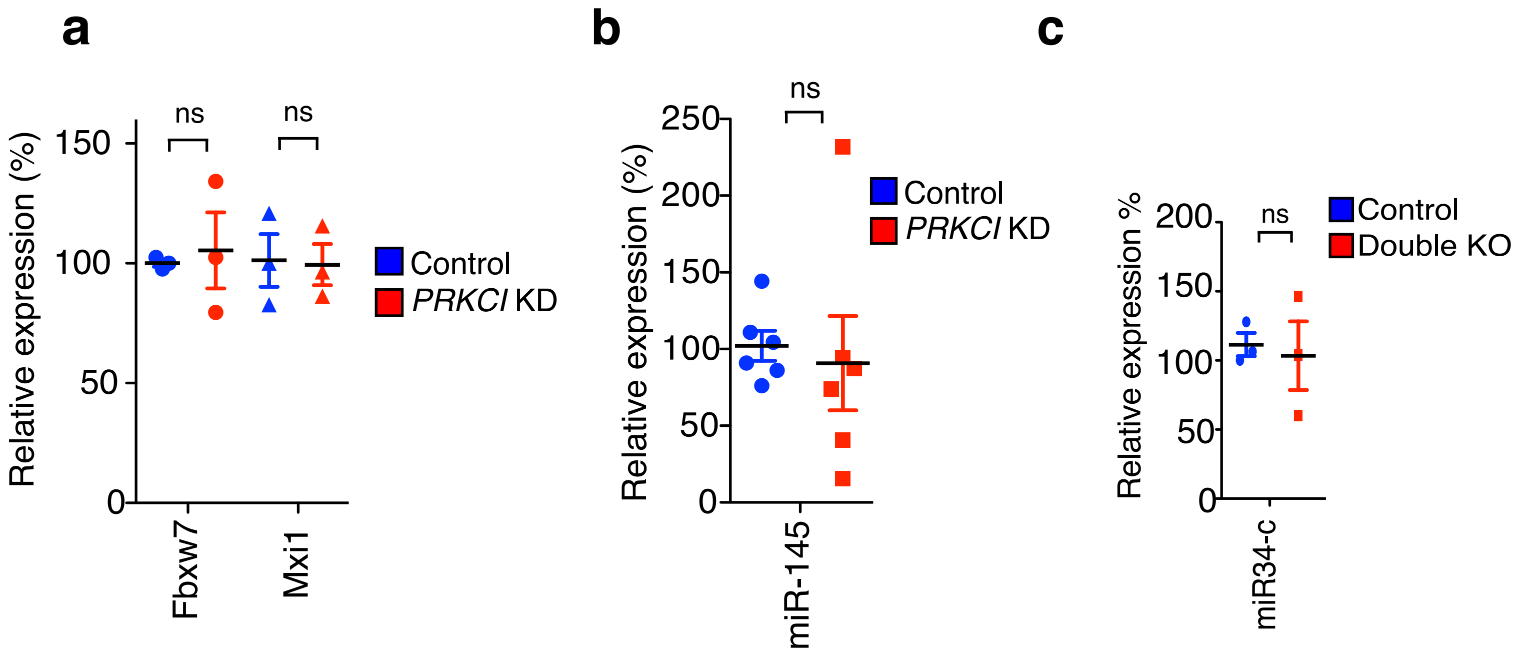

Supplementary Figure 5: a) Relative expression of known FoxO1 dependent c-Myc regulating genes *Fbxw7* and *Mxi1* in HUVEC treated with *PRKCI* siRNA or scrambled control measured by RT-PCR analysis. Data represent mean +/- S.E.M., two-tailed unpaired *t*-test; ns= $p>0.05$ $n\geq3$. **(b)** Relative expression of miR145 in HUVEC treated with siRNA targeting *PRKCI* or scrambled control measured by RT-PCR analysis. Data represent mean +/- S.E.M., two-tailed unpaired *t*-test; ns= $p>0.05$; $n\geq3$. (**c**) Relative expression of miR34-c in P6 retina of control or *Prkci/FoxO1* double knockout mice measured by RT-PCR analysis. Data represent mean +/- S.E.M., two-tailed unpaired *t*-test; ns= $p>0.05$; $n\geq3$.

Supplementary Figure 6

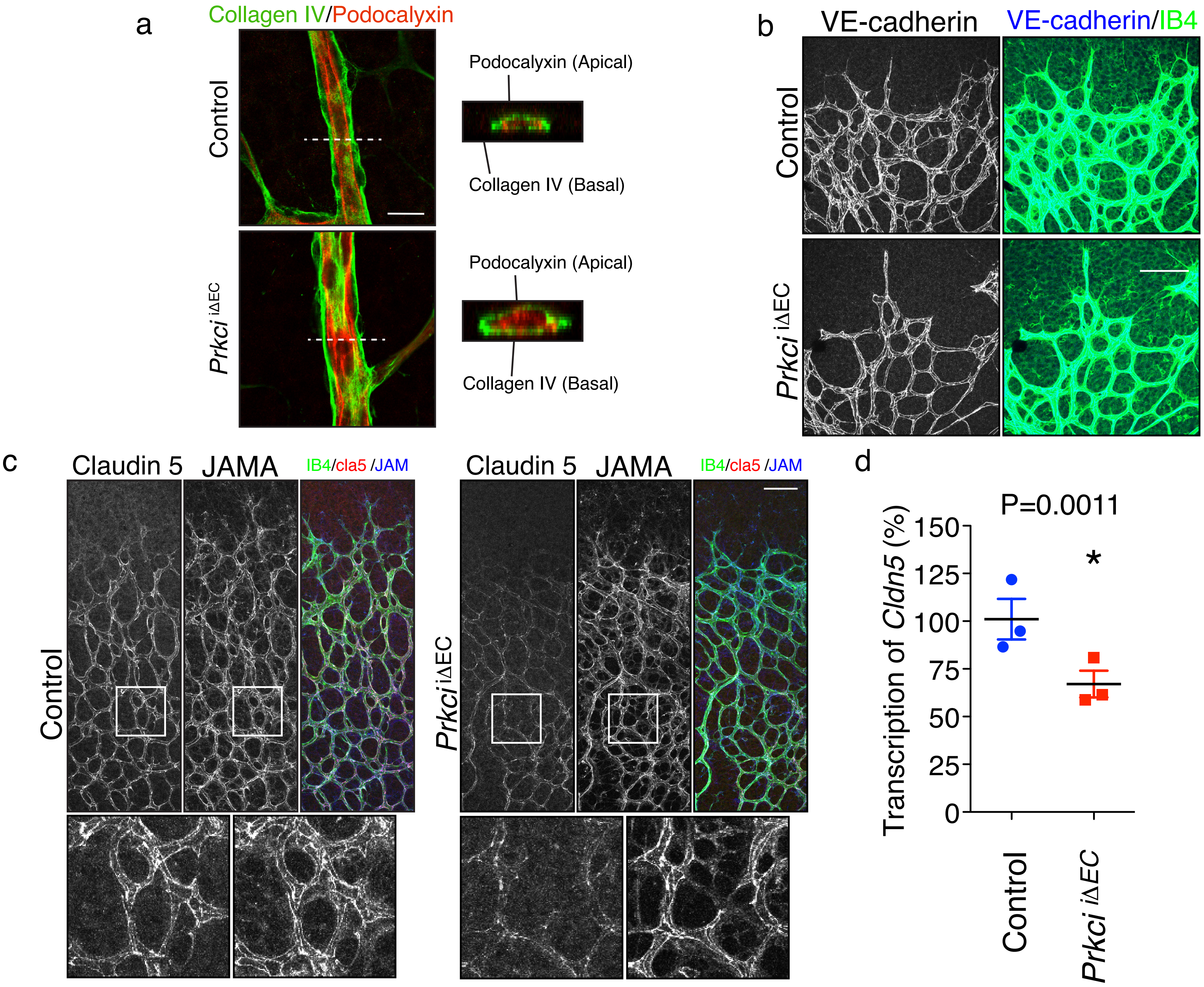

Supplementary Figure 6: (a) Podocalyxin (apical marker; red), Collagen IV (basal marker; green) staining in *Prkci*$^{i\Delta EC}$ and control P6 retina. Cross-sectional view of z-stack images from the location indicated with line on each vessel are shown on the right. Staining is representative of all vascular types within the retina observed in 3 control and 3 mutant mice. Scale bar represents 8 µm. **(b)** Staining of adherens junction protein VE-cadherin (blue) and isolectin-B4 (green) in *Prkci*$^{i\Delta EC}$ and control P6 retina. Scale bar represents 100 µm. **(c)** Staining of tight junction proteins claudin 5 (red), JAMA (blue), and isolectin-B4 in *Prkci*$^{i\Delta EC}$ and control P6 retina. Bottom panel are higher magnification of areas indicated in squares in upper panels. Staining is representative of that observed in >3 control and mutant mice. Scale bar represents 80 µm. **(d)** Relative expression of claudin 5 as analyzed by RT-PCR from *Prkci*$^{i\Delta EC}$ and control P6 retina; mean +/- S.E.M.; two-tailed students t-test; *=$p<0.05$; $n \geq 3$.

Supplementary Figure 7

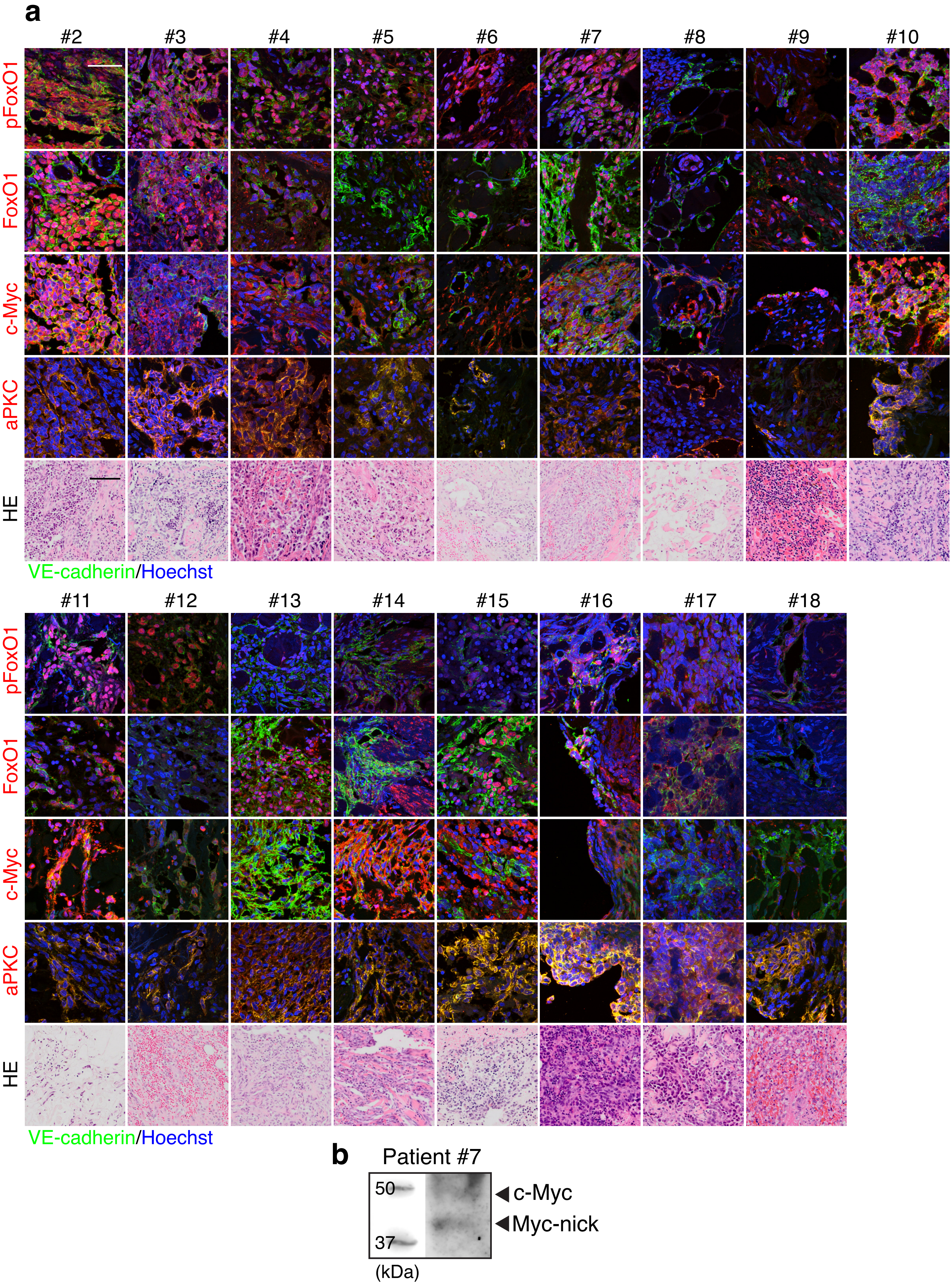

Supplementary Figure 7: (**a**) Immunofluorescent staining with anti-c-Myc, anti-FoxO1, anti-aPKC, or pSer218-FoxO1 antibodies (red) and anti-VE-cadherin (green) with Hoechst 33342 (blue) in angiosarcoma tissue samples. Corresponding haematoxylin and eosin staining for each patient (bottom panel). Fluorescent images scale bar represents 50 µm; H&E images scale bar represents 100 µm. **(b)** Western blot analysis of c-Myc expression from tissue sections of Patient #7. Arrowheads indicate c-Myc and the cytoplasmic cleavage product Myc-nick.

Supplementary Figure 8

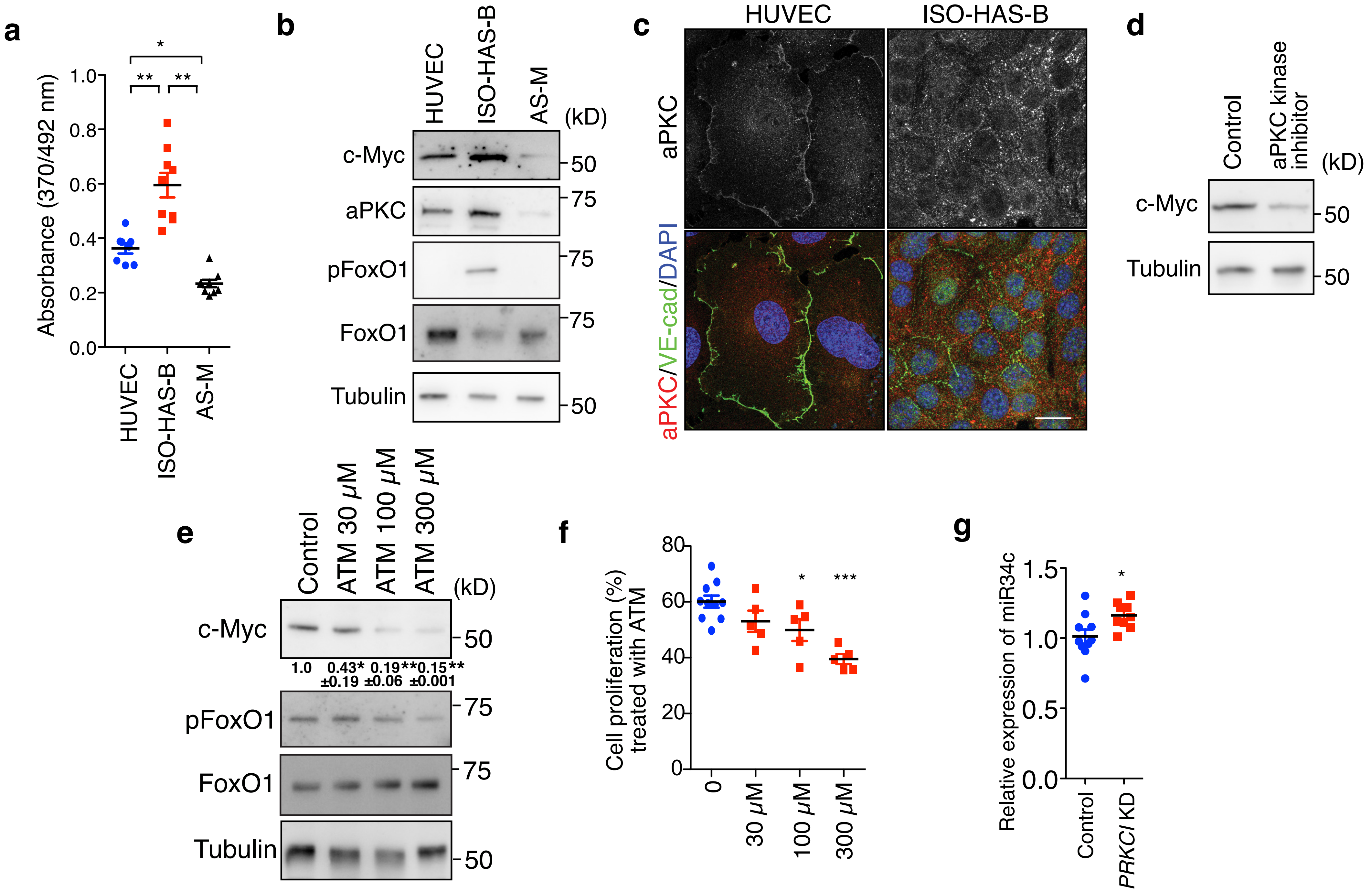

Supplementary Figure 8: aPKCλ is important for angiosarcoma cell c-Myc expression and proliferation. **(a)** Relative BrdU incorporation in HUVEC compared to tumorogenic ISO-HAS-B patient derived angiosarcoma cells and non-tumorgenic AS-M patient derived angiosarcoma cells; mean +/- S.E.M.; one-way ANOVA with Bonferroni post-hoc analysis; *=p<0.05; **=p<0.01 (*n=8 or 9)*. **(b)** Western blot analysis of c-Myc and aPKC abundance in HUVEC and angiosarcoma cell lines. **(c)** Immunofluorescent staining of aPKC (red) VE-Cadherin (green) and DAPI in HUVEC and ISO-HAS-B cells. Scale bar represents 20 µm. **(d)** Western blotting analysis of c-Myc expression after treatment of ISO-HAS-B cells with aPKC kinase inhibitor. **(e)** Western blotting analysis of c-Myc expression after treatment of ISO-HAS-B cells with aPKC inhibitor ATM; Densitometric quantifications are shown below the lanes mean +/- S.E.M.; one-way ANOVA with Bonferroni post-hoc analyses; *=p<0.05; **p=<0.01; (*n*=3). **(f)** Relative BrdU incorporation in ISO-HAS-B cells after treatment with ATM for 24 hours. Data represent mean ± S.E.M. one-way ANOVA with Bonferroni post-hoc analysis *=p<0.05; ***=p<0.001 (*n*=5). **(g)** Expression of miR-34c in ISO-HAS-B cells after treatment with *PRKCI* or control siRNA mean +/- S.E.M.; two-tailed unpaired *t*-test; *=p<0.05 (*n=9 or* 10).

Supplementary Figure 9

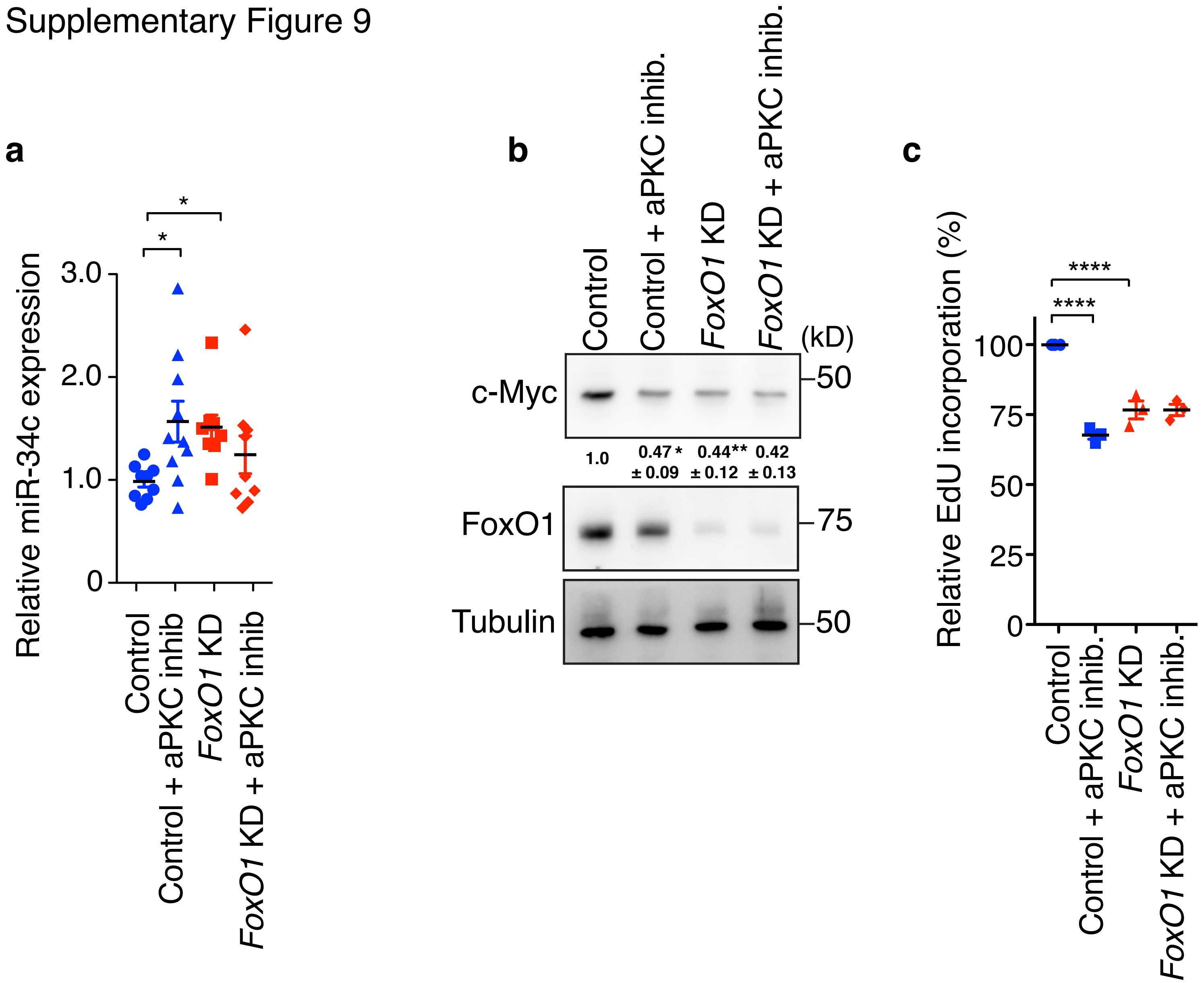

Supplementary Figure 9: FoxO1 KD in ISO-HAS-B cells blocks the effect of aPKC inhibition on miR-34c expression. **(a)** Expression of miR-34c in ISO-HAS-B cells after treatment with FoxO1 siRNA or scrambled control with and without ATG treatment. Data represents mean +/- S.E.M.; Two-way ANOVA with Bonferroni post-hoc analyses; *=p<0.05 vs. control untreated; (*n*=9). **(b)** Western blot analysis of c-Myc and FoxO1 in ISO-HAS-B cells after treatment with FoxO1 siRNA or scrambled control with and without aPKC kinase inhibitor treatment. Densitometric quantifications are shown below the lanes mean +/- S.E.M.; Two-way ANOVA with Bonferroni post-hoc analyses; *=p<0.05 vs. control untreated; **=p<0.01 vs. control untreated (*n*=3). **(c)** Relative EdU incorporation in ISO-HAS-B cells after treatment with FoxO1 siRNA or scrambled control with and without aPKC kinase inhibitor treatment. Data represents mean +/- S.E.M.; Two-way ANOVA with Bonferroni post-hoc analyses; ****=p<0.0001 vs. control untreated (n=3).

Riddell et al., Supplementary Fig. 10

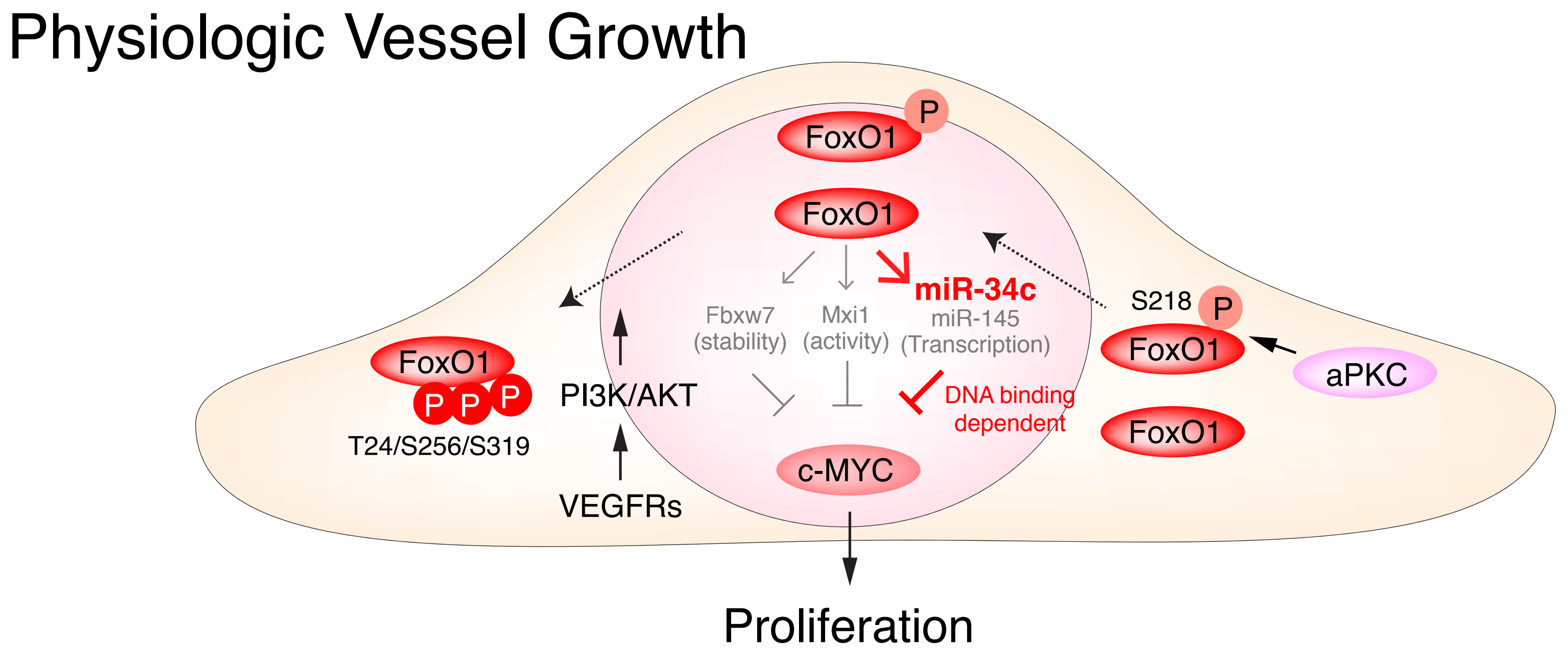

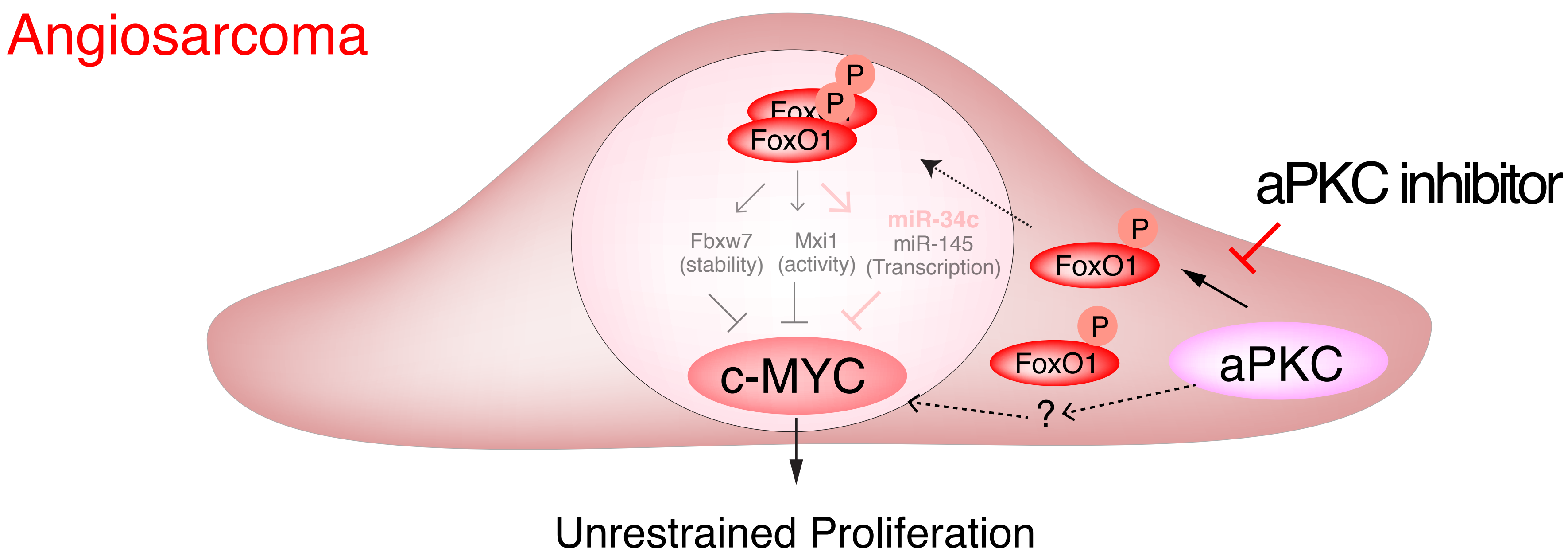

Supplementary Figure 10: Schematic diagram of aPKC/FoxO1/c-Myc signaling pathway. aPKC controls physiological (top panel) and pathological growth (bottom panel) by regulating the transcriptional activity and abundance of key transcription factors FoxO1 and c-Myc respectively. Top panel: The PI3K/Akt pathway controls c-Myc expression by regulating the phosphorylation of FoxO1 at Thr24/Ser256/Ser319, which results in cytoplasmic sequestration of FoxO1 and moderate expression of c-Myc. As a second mechanism controlling c-Myc expression, aPKC directly phosphorylates FoxO1 at Ser218 within the DNA binding domain, which results in reduced transcription of the miR34-c gene and moderate expression of c-Myc. Bottom panel: In angiosarcoma cells, high levels of aPKC are likely to result in enhanced FoxO1 Ser218 phosphorylation and strongly reduced expression of miR34-c. APKC may also contribute to c-Myc expression through another unknown mechanism. As a consequence, c-Myc is highly expressed leading to unrestrained proliferation. Inhibition of aPKC could provide a promising approach to control angiosarcoma cell proliferation in patients.

Supplementary Figure 11

Figure 7a- anti-c-Myc

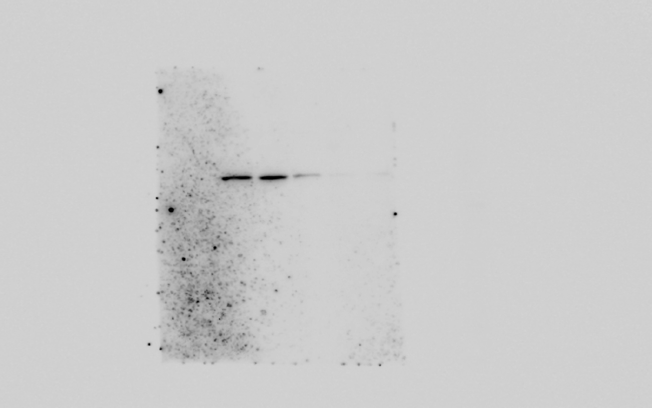

Figure 7a- anti-pSer218 FoxO1

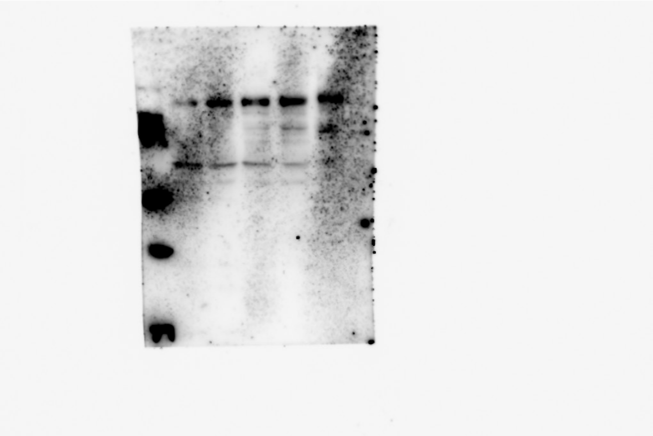

Supplementary Figure 1a- anti-c-Myc (9E10 antibody (Sc-40))

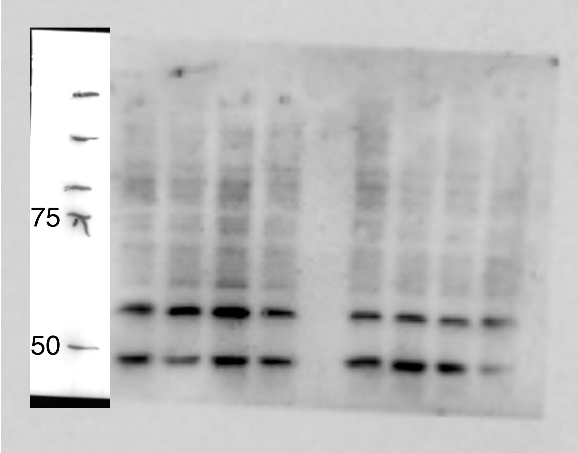

Supplementary Figure 7b- anti-c-Myc (Millipore 06-340)

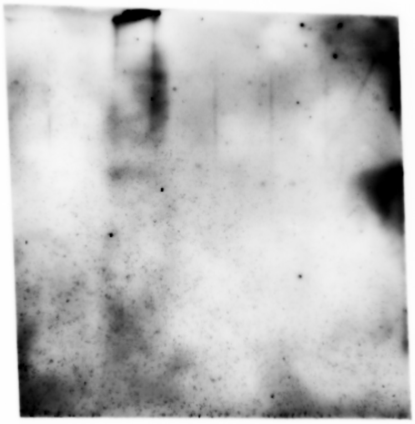

Supplementary Figure 11: Uncropped western blots from indicated figures.

Supplementary Table 1:

Patient Number	Age	Sex	Primary Tumor	C-Myc	FoxO1		aPKC	Follow up (Months)	Alive
				Expression	Expression	pSer218	Expression		
#1	68	F	Liver	++	++	Positive	+++	-	
#2	61	M	Scalp	+++	+++	Positive	++	9	
#3	75	M	Scalp	++	++	Positive	+++	34	
#4	76	M	Scalp	++	+	Positive	+++	8	
#5	79	F	Scalp	+	±	Positive	++	-	
#6	66	M	Scalp	+	+	Positive	++	35	
#7	71	F	Leg	++	++	Positive	++	22	
#8	89	F	Scalp	+	±	Negative	+	14	
#9	79	M	Scalp	+	+	Negative	+	32	
#10	88	F	Scalp	+++	+	Positive	+++	15	
#11	51	F	Leg	++	++	Positive	+	78	yes
#12	88	F	Scalp	+	±	Positive	+	14	
#13	68	M	Scalp	+	++	Negative	++	66	yes
#14	34	M	Scalp	+++	++	Negative	++	17	
#15	91	M	Scalp	++	++	Negative	++	12	
#16	81	M	Scalp	+	++	Positive	+++	11	
#17	79	M	Scalp	±	+	Positive	++	9	
#18	78	M	Scalp	±	±	Positive	++	15	
#19	20	M	Shoulder	NE	NE	Negative	+	70	yes
#20	39	F	Thigh	NE	NE	Positive	+	9	
#21	74	M	Temple	NE	NE	Positive	+	6	
#22	57	F	Arm	NE	NE	Negative	++	4	
#23	60	M	Scalp	NE	NE	Negative	+	96	

#24	79	F	Breast skin	NE	NE	Positive	++	83	yes
#25	78	F	Scalp	NE	NE	Positive	++	5	
#26	71	M	Scalp	NE	NE	Positive	++	6	
#27	72	F	Scalp	NE	NE	Positive	++	30	
#28	76	F	Abdomen	NE	NE	Positive	+	10	
#29	80	M	Scalp	NE	NE	Positive	+++	7	yes
#30	80	M	Scalp	NE	NE	Positive	++	3	
#31	88	F	Leg	NE	NE	Positive	+++	12	
#32	70	M	Scalp	NE	NE	Positive	+	39	
#33	74	M	Scalp	NE	NE	Positive	++	23	
#34	90	F	Scalp	NE	NE	Positive	++	18	
#35	72	M	Scalp	NE	NE	Positive	+	6	
#36	68	M	Scalp	NE	NE	Negative	+	78	
#37	72	F	Scalp	NE	NE	Negative	+	59	yes
#38	82	F	Scalp	NE	NE	Negative	+	25	yes
#39	91	M	Scalp	NE	NE	Positive	+	11	yes

weak, + moderate, ++ strong, +++ very strong, NE not examined

Supplementary Table 2:

ON-TARGET plus Human FOXO1 siRNA target sequences:
GCGCUUAGACUGUGACAUG
GAGGUAUGAGUCAGUAUAA
UGACUUGGAUGGCAUGUUC
GGACAACAACAGUAAAUUU

Supplementary Table 3:

CDK4 Fwd	TCTGGTGACAAGTGGTGGAAC
CDK4 Rev	TGGTCGGCTTCAGAGTTTCC
Cyclin B2 Fwd	GGAAGTCATGCAGCACATGG
Cyclin B2 Rev	TCCTATCAGTGGGGAGGCAA
Enolase-1 Fwd	CATCAATAAAACTATTGCGCCTGC
Enolase-1 Rev	CCCCCAGAATGGCGTTCG
FASN Fwd	TATGAAGCCATCGTGGACGG
FASN Rev	GAAGAAGGAGAGCCGGTTGG
LDHB Fwd	GCGGAGAGACTTGTTGCATT
LDHB Rev	AATGCTGATAGCACACGCCA
Cyclin D1 Fwd	GATGCCAACCTCCTCAACGA
Cyclin D1 Rev	GGAAGCGGTCCAGGTAGTTC
LDHA Fwd	GTCAGCATAGCTGTTCCACTTA
LDHA Rev	AGCTGATCCTTTAGAGTTGCCA
Cyclin D2 Fwd	GCTCACTTGTGATGCCCTGA
Cyclin D2 Rev	CGGTACTGCTGCAGGCTATT
B2M Fwd	CACCCCCACTGAAAAAGATGAG
B2M Rev	CCTCCATGATGCTGCTTACATG
$p27^{kip1}$ ChIP RT Fwd	GTACAGATCTCCGGTGCCTT
$p27^{kip1}$ ChIP RT Rev	AGTCACAGTCACACCACCTT
Cyclin D1 ChIP RT Fwd	GGACGTCTACACCCCCAACA
Cyclin D1 ChiP RT Rev	CCTTCCTACCTTGACCAGTC
miR-34c ChiP RT Fwd	ACACTCTTCTTGGCTTCCTCC
miR-34c ChiP RT Rev	AAGGAAGGCACCTTTCAGGG

Supplementary Table 4:

Assay Name	Assay ID
hsa-miR-16-5p	477860_mir
has-miR145-5p	477916_mir
hsa-miR-186-5p	477940_mir
hsa-miR-34c-5p	478052_mir
Gapdh	Mm99999915_g1
Cldn5	Mm00727012_s1
